# Brensocatib – an editorial on the new chapter in noncystic fibrosis bronchiectasis treatment

**DOI:** 10.1097/MS9.0000000000003976

**Published:** 2025-09-30

**Authors:** Muhammad Furqan, Muhammad Usman Haider, Sadia Binte Rahim

**Affiliations:** a Department of Medicine, King Edward Medical University, Lahore, Pakistan; b Department of Internal Medicine, Geisinger Health System, Wilkes-Barre, Pennsylvania, USA; c Department of Medicine, Mymensingh Medical College Hospital, Bangladesh

**Keywords:** brensocatib, bronchiectasis, dipeptidyl peptidase-1 (DPP-1), neutrophil serine proteinases (NSPs), pulmonary exacerbations

## Abstract

Bronchiectasis is a chronic airway disease marked by irreversible bronchial dilation, persistent cough, and recurrent infections. Its pathogenesis is explained by the “vicious cycle hypothesis,” which involves impaired mucociliary clearance, neutrophil activation, and tissue damage from neutrophil serine proteinases (NSPs). In August 2025, the FDA approved Brensocati, a selective dipeptidyl peptidase-1 (DPP-1) inhibitor, as the first disease-modifying therapy for bronchiectasis. By blocking NSP activation, Brensocatib reduces inflammation and exacerbation. WILLOW and ASPEN trials demonstrated significant improvements in exacerbation rates, lung function decline, and exacerbation-free survival, establishing a novel therapeutic paradigm for this previously undertreated condition.

Bronchiectasis is characterized by the abnormal dilation and thickening of the bronchial wall on lung imaging. It manifests primarily through persistent cough and excessive sputum production^[1]^. The common etiologies of bronchiectasis include cystic fibrosis. In contrast, non-cystic fibrosis bronchiectasis (NCFB) commonly arises from lung infections (most common), bronchial obstruction, postinflammatory pneumonitis, and pulmonary diseases such as COPD^[2]^. The pathophysiology of NCFB is not well established, but Cole^[3]^ described it as a “vicious cycle hypothesis.” This self-perpetuating cycle starts with a primary insult, e.g., an infection that hampers the mucociliary clearance, enhancing the chances of colonization of pathogens in the respiratory tract. This infection leads to inflammation predominantly by activating neutrophils^[4]^. The activated neutrophils release various mediators, including neutrophil elastase, proteinase 3, and cathepsin G, collectively termed neutrophil serine proteinases (NSP). NSP weakens the bronchial wall and results in permanent bronchial dilation and loss of lung function^[5]^. The inflammation further worsens the mucociliary impairment. This cycle carries on, and the damage becomes irreversible^[4]^.

**HIGHLIGHTS**

- FDA approval of Brensocatib in August 2025 as first therapy for bronchiectasis.
- Selective DPP-1 inhibition reduces neutrophil-driven airway inflammation.
- ASPEN and WILLOW trials showed fewer exacerbations and slower lung function decline.
- Adverse events include respiratory infections, hyperkeratosis, and periodontal disease.
- Marks a shift from symptomatic relief to disease-modifying treatment.

The management of bronchiectasis has mainly relied on symptomatic treatment. However, in August 2025, the U.S. Food and Drug Administration (FDA) approved Brensocatib as the first and only treatment for bronchiectasis in adults and children aged ≥12 years^[6]^. Brensocatib is a novel, orally administered small molecule that acts as a selective, competitive, and reversible inhibitor of dipeptidyl peptidase-1 (DPP-1)^[7]^. DPP-1 is a cystein protease that plays a significant role in the activation of NSPs. NSPs are produced as inactive pro-proteins in the bone marrow and require DPP-1 mediated trimming of their N-terminal peptide to become active before being packaged into the granules^[8]^. Brensocatib inhibits DPP-1, which reduces NSP activity, attenuates airway inflammation, and offers a novel therapeutic strategy for bronchiectasis. The approval of Brensocatib by the FDA is a landmark step in treating bronchiectasis, and this approval is backed by the phase 3 ASPEN trial and phase 2 WILLOW trial^[6]^.

The ASPEN trial was a 52-week, phase 3, double-blinded, randomized, placebo-controlled trial that investigated the safety, efficacy, and tolerability of Brensocatib in 1721 patients with NCFB. The primary end point of the trial was the confirmed number of pulmonary exacerbation events per year during the 52-week treatment period, i.e., the annualized rate of adjudicated pulmonary exacerbations^[7]^. The results demonstrated that the annualized rate were significatly lower with both 10 mg dose [annualized rate = 1.02 (95% confidence interval (CI), 0.91–1.13; rate ratio (RR): 0.79; adjusted *P* = 0.004)] and 25 mg dose [annualized rate = 1.04 (95% CI, 0.93–1.16; RR: 0.81; adjusted *P* = 0.005) as compared to the placebo [rate = 1.29 (95% CI, 1.16–1.43)]. Secondary outcomes showed that the Brensocatib group had a longer time to first exacerbation, and exacerbation-free time was also higher in this group. The decline in lung function, as measured by forced expiratory volume in 1 s (FEV_1_), was slower in the 25 mg dose group compared with placebo^[7]^.

The findings of the phase 3 ASPEN trial are consistent with the phase 2 WILLOW trial. This 24-week, double-blinded, randomized, placebo-controlled study enrolled 256 patients with bronchiectasis who had experienced at least two exacerbations in the previous year^[9]^. Participants were randomized in a 1:1:1 ratio to placebo, 10 mg Brensocatib, or 25 mg Brensocatib. The trial reported that the time to first exacerbation (primary endpoint) was prolonged with both 10 mg [hazard ratio (HR): 0.58; 95% CI, 0.35–0.95, *P* = 0.03) and 25 mg (HR: 0.62; 95% CI, 0.38–0.99, *P* = 0.046) Brensocatib compared with placebo. Furthermore, the overall rate of exacerbation was also lower in the Brensocatib groups as compared to the placebo. These significant improvements in the primary and secondary endpoints contributed to the accelerated FDA approval of Brensocatib^[9]^.

However, Brensocatib is not devoid of side effects. Among the treatment-related adverse events (TRAEs), COVID-19, cough, headache, nasopharyngitis, dyspnea, and diarrhea were most commonly reported^[7,9]^. While the most common serious TRAEs included pulmonary exacerbations and pneumonia. TRAEs of special interests included hyperkeratosis and peridontal disease (peridontitis or gingivitis), which are features of Papillon–Lefèvre syndrome, a condition caused by a mutation in DPP-1 and near-complete loss of DPP-1 function^[7,9]^. These adverse events, particularly the risks of hyperkeratosis and periodontal disease, formed the basis for the inclusion of a boxed warning to alert healthcare professionals to the potential for clinically significant inflammatory and dental complications associated with Brensocatib therapy.

Based on the findings of the ASPEN and WILLOW trials, Brensocatib represents a significant advancement in the management of bronchiectasis by addressing the underlying neutrophil-driven inflammation rather than offering only symptomatic relief. Although long-term safety and real-world effectiveness require further evaluation in broader patient populations, it has shown dose-dependent efficacy. It reduces exacerbation frequency, prolongs the time to first exacerbation, slows lung function decline, and increases exacerbation-free survival. Its once-daily oral dosing also offers a convenient alternative to inhaled or intravenous therapies. This novel approach opens the door to a new treatment paradigm for patients with this chronic and previously undertreated condition.

## Data Availability

The datasets are available from the corresponding author on reasonable request.

## Research registration unique identifying number (UIN)

This is not an original research project involving human participants in an interventional or an observational study but a case report. This registration was not required.

## AI use declaration

To ensure transparency, this work complies with the TITAN 2025 guidelines (Transparency In The reporting of Artificial INtelligence) (10). All AI use has been declared and reviewed in line with these recommendations. Generative AI was employed only for grammar, spelling, and clarity improvements during drafting and revision, without involvement in data analysis or figure creation. Grammarly (web-based, version 14.1249.0) and ChatGPT (OpenAI GPT-5 via chat.openai.com, default settings) were used exclusively for language refinement, with no plug-ins, fine-tuning, or integrations. No patient text or images were used; all inputs complied with GDPR/HIPAA. Outputs were reviewed, edited, and approved by the supervising author, who assumes full responsibility for the final content. AI tools were applied solely for language refinement, using simple text-based prompts, with full adherence to ethical standards.
